# Lamotrigine-Loaded Poloxamer-Based Thermo-Responsive Sol–Gel: Formulation, In Vitro Assessment, Ex Vivo Permeation, and Toxicology Study

**DOI:** 10.3390/gels9100817

**Published:** 2023-10-14

**Authors:** Maria Riaz, Muhammad Zaman, Huma Hameed, Hafiz Shoaib Sarwar, Mahtab Ahmad Khan, Ali Irfan, Gamal A. Shazly, Ana Cláudia Paiva-Santos, Yousef A. Bin Jardan

**Affiliations:** 1 Faculty of Pharmaceutical Sciences, University of Central Punjab, Lahore 54000, Pakistan; 2 Department of Chemistry, Government College University Faisalabad, Faisalabad 38000, Pakistan; raialiirfan@gmail.com; 3 Department of Pharmaceutics, College of Pharmacy, King Saud University, Riyadh 11451, Saudi Arabia; 4 Department of Pharmaceutical Technology, Faculty of Pharmacy of the University of Coimbra, University of Coimbra, 3000-548 Coimbra, Portugal; 5 REQUIMTE/LAQV, Group of Pharmaceutical Technology, Faculty of Pharmacy, University of Coimbra, 3000-548 Coimbra, Portugal

**Keywords:** lamotrigine, epilepsy, poloxamer 407, nose to brain, intranasal

## Abstract

The present study aimed to prepare, characterize, and evaluate a thermo-responsive sol–gel for intranasal delivery of lamotrigine (LTG), which was designed for sustained drug delivery to treat epilepsy. LTG sol–gel was prepared using the cold method by changing the concentrations of poloxamer 407 and poloxamer 188, which were used as thermo-reversible polymers. The optimized formulations of sol–gel were analyzed for clarity, pH, viscosity, gelation temperature, gelation time, spreadability, drug content, in vitro drug release studies, ex vivo permeation studies, and in vivo toxicological studies. FTIR, XRD, and DSC were performed to determine the thermal stability of the drug and polymers. The prepared formulations had a clear appearance in sol form; they were liquid at room temperature and became gel at temperatures between 31 °C and 36 °C. The pH was within the range of the nasal pH, between 6.2 and 6.4. The drug content was found to be between 92% and 94%. In vitro drug release studies indicated that the formulations released up to 92% of the drug within 24 h. The FTIR, DSC, and XRD analyses showed no interaction between the drug and the polymer. A short-term stability study indicated that the formulation was stable at room temperature and at 4–8 °C. There was a slight increase in viscosity at room temperature, which may be due to the evaporation of the vehicle. A histological study indicated that there were no signs of toxicity seen in vital organs, such as the brain, kidney, liver, heart, and spleen. It can be concluded from the above results that the prepared intranasal sol–gel for the delivery of LTG is safe for direct nose-to-brain delivery to overcome the first-pass effect and thus enhance bioavailability. It can be considered an effective alternative to conventional drug delivery for the treatment of epilepsy.

## 1. Introduction

Over the past few years, in situ gel systems have appeared as a novel approach for drug release in a sustained and controlled way due to their distinctive property of ‘Sol to Gel’ transformation. The gel is the transitional stage between the solid and liquid phases. The liquid phase is immobilized by the solid component, which is made up of a three-dimensional network of interconnected molecules [1]. At room temperature, sol–gel is in a solution form and converts into a gel form under body conditions [2]. There are various mechanisms that can cause in situ gels to form, including those that are based on physiologic stimuli (such as temperature changes or pH-triggered systems) [3,4], those based on physical changes in biomaterials (such as solvent exchange and swelling) [5], and those that are based on chemical reactions (such as UV radiation, ionic crosslinking, and ion-activated systems) [6,7]. In thermo-reversible sol–gel polymers, temperature-sensitive properties are being used [8]. Thermo-responsive or temperature-responsive polymers show an abrupt change in their physical properties with a temperature change. Temperature-sensitive sol–gel is formulated in this study. The most important and effective polymer for this approach is poloxamer 407. Using this method, gel forms at body temperature. These in situ gels are liquid at 25 °C, but when they come into contact with bodily fluids at 35 to 37 °C, they turn into gel [9].

**Figure 1:**
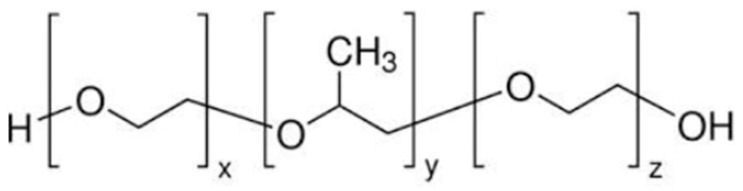
Structure of poloxamer 407 [10].

Poloxamer is a commercially available polymer with temperature-sensitive properties. It consists of two hydrophilic units of polyethylene oxide and a central hydrophobic unit of propylene oxide, making it water soluble. Poloxamer can make transparent and clear gels. In a concentrated solution, poloxamer forms thermo-reversible gels. Poloxamer has different grades, such as poloxamer 124, 188, 237, 338, and 407. Poloxamer can be used as an emulsifying agent, gelling agent, and solubility-enhancing agent. The structure of poloxamer 407 is shown in Figure 1 [10,11]. These gels are used for delivering poorly water-soluble medicines because poloxamer 407 generates micelles and can solubilize hydrophobic molecules. The micellization and solubilization characteristics of poloxamer 407 must therefore be understood [12]. We use poloxamer 407’s thermo-reversible behavior to formulate intranasal sol–gel; both hydrophobic and hydrophilic groups present in this polymer allow lamotrigine to be soluble at nasal pH [13,14]. Poloxamer 188 is used along with poloxamer 407 to optimize the sol-to-gel to target a specific range of temperatures [15].

The intranasal route is used for targeted delivery in this study. Nose-to-brain delivery is a non-invasive technique that allows drugs to be delivered to the CNS while avoiding the BBB by going straight to the brain [16,17]. The nasal cavity present in the nose is further divided into the respiratory area and the olfactory area for medication administration. The surface and structural characteristics of the supplied biomolecules, such as lipophilicity, size, and degree of ionization, have a substantial impact on nose-to-brain transport. Anatomically, the placement of the olfactory epithelium in the roof of the nasal cavity makes it challenging to deliver drugs to the desired location. There are three different potential routes for drugs to cross the olfactory epithelium: (a) a paracellular pathway through the use of tight junctions present among both sustentacular cells and olfactory neurons; (b) a transcellular pathway, primarily across the sustentacular cells, most commonly through endocytosis mediated by receptors, liquid endocytosis, or passive transport (not for peptides); and (c) the olfactory nerve route, where the medication enters the neurons through endocytotic or pinocytotic processes and travels to the olfactory bulb via intracellular axonal transport [18,19]. The hypothalamus, olfactory tract, amygdala, entorhinal cortex, anterior olfactory nucleus, etc. are only a few of the areas of the brain to which the olfactory bulbs project. Upon intranasal medication administration, these projections facilitate intra- and peri-neural trafficking. The trigeminal nerves, which include branches like the ophthalmic, maxillary, and mandibular nerves, project into the nasal cavity to supply the respiratory region with nerves. These branches create access into the rostral and caudal areas of the brain by entering the cribriform plate and the lacerated foramen [20]. By using the olfactory or trigeminal nerves situated in the upper section of the nasal canal, the medications can quickly enter the CNS through a “shortcut” from the nose to the brain. Reduced plasma exposure, fewer side effects in the peripheral area, prevention from hepatic first-pass metabolism, and protection from the harmful effects of gastric acid are all significant benefits of direct drug delivery from the nose to the brain [18,21].

**Figure 2:**
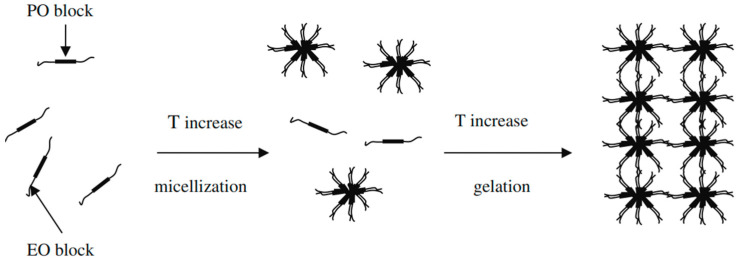
Diagrammatic representation of poloxamer 407 micelle formation [15].

Lamotrigine belongs to BCS class II and has poor solubility and high permeability. According to reports, lamotrigine dissolves in water at 0.17 mg mL^−1^ at 25 °C (The Internet Drug Index, 2007). Although lamotrigine has gained widespread acceptance in the treatment of seizures, its poor aqueous solubility limits its absorption and dissolution rate and thus delays the onset of action (Hurley 2002) [22]. To enhance the solubility of drugs with poor solubility, different novel techniques for drug delivery have been developed, like the reduction of the particle size, the solid dispersion technique, microparticles, nanotechnology, etc. [23]. Poloxamer is used in this study to formulate an LTG sol–gel that, along with its thermo-responsive properties, enhances the solubility of LTG through micellization as shown in Figure 2.

The difficulties of frequent medication administration and significant changes in drug blood levels are common problems with drug delivery in conventional dose formulations. This study aims to develop a novel drug delivery system that will directly target the brain to treat epilepsy. This will prevent first-pass metabolism. It will improve the drug’s pharmacokinetic profiles by improving brain targeting through the olfactory area due to the favorable anatomical features of the nasal epithelium. Lamotrigine sol–gel given intranasally is in solution form at room temperature and converted into a gel when it comes into contact with the nasal mucosa, resulting in a decrease in drug release frequency and, hence, an increase in bioavailability. This targeted drug delivery system reduces local and systemic adverse effects associated with LTG by preventing the exposure of other tissues and cells to the drug.

## 2. Results

### 2.1. Formulation Optimization Data Analysis

After the initial investigations, the values of three coded levels of two components were assumed, as shown in Table 1.

**Figure 3:**
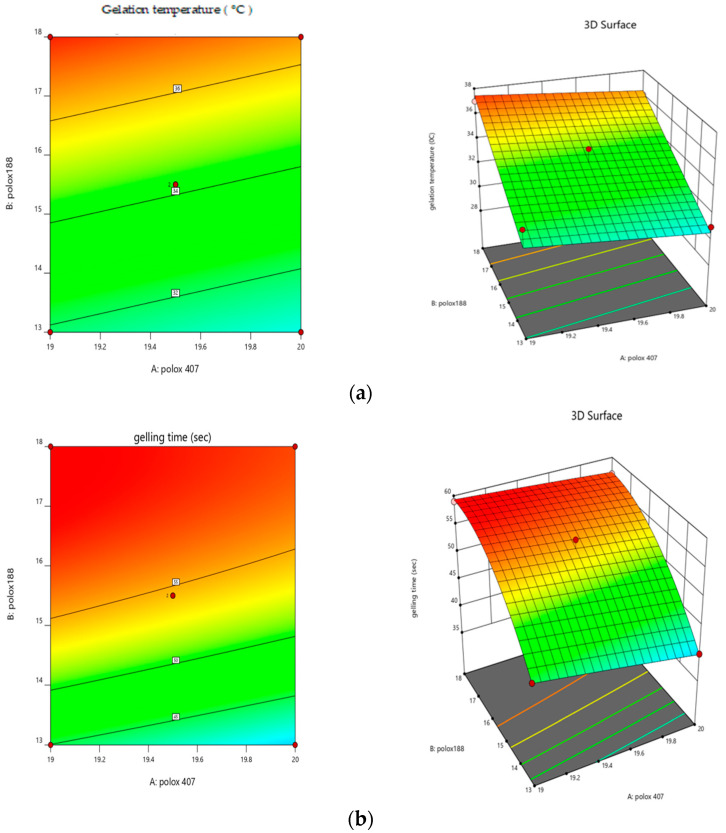
Response surface plots showing (**a**) the effects of poloxamer 407 and 188 on gelation temperature and (**b**) the effects of poloxamer 407 and 188 on gelation time.

Significant model probability was shown by the RSM data analysis at a *p*-value less than 0.05 and significant for both gelation temperature and gelling time as shown in Figure 3. Polynomial Equation (2) showed that poloxamer 407 had an inverse relationship with the gelation temperature as the concentration of poloxamer 407 increased and the gelation temperature decreased. Poloxamer 188 had a direct relationship with the gelation temperature. As the concentration of poloxamer 188 increased, the gelation temperature also increased. Polynomial Equation (3) showed that poloxamer 407 had an inverse relationship with the gelation time, as the concentration of poloxamer 407 increased as the gelation time decreased. Poloxamer 188 had a direct relationship with the gelation time. As the concentration of poloxamer 188 increased, the gelation time also increased. The analysis of the responses, such as gelation temperature and gelling time, revealed the effects of the thermo-reversible polymers employed. The R2 for the gelation temperature was 0.89, and the R2 for the gelling time was 0.99, demonstrating a useful correlation coefficient between the models, as shown in Table 2.

The effect of the polymers used (poloxamer 407 and poloxamer 188) was seen in relation to the gelation temperature and the gelling time using a polynomial equation, which is shown below in Equations (2) and (3).

Gelation temperature = 34 β_0_ − 0.05 X_1_+ 2.89 X^2^ + 0.68 X_1_X_2_ − 0.05 X_1_^2^ − 3.4 X_2_^2^ (1)

Gelling time = 54 β_0_ − 1.76 X_1_+ 7.94 X^2^ + 0.75 X_1_X_2_ − 0.0625 X_1_^2^ − 4.06 X_2_^2^ (2)

### 2.2. Clarity

The clarity of all three selected formulations was assessed visually against a light and dark background. At room temperature, all of the developed formulations were clear, free-flowing liquids that included no particles.

### 2.3. pH

In Table 3, the pH of all of the selected formulations is displayed. Each formulation had a pH between 5.5 and 6.5, which is the range of the nasal cavity [24].

### 2.4. Viscosity

The viscosity of the selected formulations ranged from 14,000 to 16,000 cps. The viscosity of the formulations increased as the concentration of poloxamer 188 increased. An optimal formulation for a nasal in situ gel should have an appropriate viscosity to enable simple spraying of the liquid form, which then undergoes a quick sol–gel transition as the temperature rises. The viscosity of the selected formulations is shown in Table 3.

### 2.5. Sol–Gel Gelling Temperature and Time Measurement

**Figure 4:**
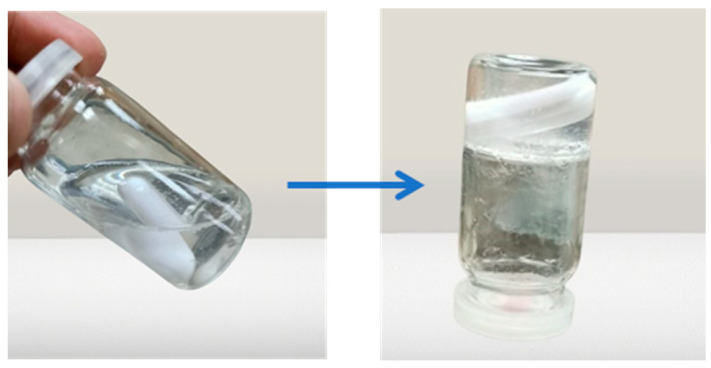
Sol to gel transition of the prepared formulation.

The sol–gel transition temperature can be interpreted as the temperature at which the phase transition of the sol meniscus is first noticed when kept in a sample tube at a specific temperature and then heated at a specific rate for in situ gel-forming systems incorporating thermo-reversible polymers. When you tilt the tube, the meniscus should not move, which is a sign that a gel has formed, as shown in Figure 4. The initial time when gelation is detected, as previously specified, is the gelling time. The gelling temperature and time of the selected formulations are given in Table 3.

### 2.6. Gel Strength

The composition of the nasal gel must have the proper gel strength, which is essential. The values for measuring the gel strength are shown in Table 3. It was discovered that the concentrations of polymers had an impact on the gel strength.

### 2.7. Drug Content

The drug was placed in the gel formulation for the current experiment. Drug uniformity before and after gelation was evaluated. The drug content before gelation of F1, F2, and F3 was found to be 93.7, 90.5, and 95, respectively. The percentage of drug content for all formulations after gelation was determined to be between 90 and 94%. There was no major change in drug content before and after gelation. Table 3 shows the percentage of drug content after gelation for all formulations.

### 2.8. Spreadability

For in situ gel to be administered with ease and to spread readily over the nasal mucosa without leaking after application, it must have the proper spreadability. The data for the spreadability measurement are displayed in Table 3. Formulation F3 had the greatest spreadability because the in situ gel covered a larger surface area.

### 2.9. Stability Studies

A stability study of the best-optimized formulation F3 was carried out in a humidity control chamber, and the temperature of the chamber was set at 25 °C ± 2 °C/65% ± 5% relative humidity and at 4 °C ± 1 °C for 3 months. Under the above-mentioned conditions, the drug’s content remained the same until the end of the third month. An in vitro investigation of drug release revealed that drug release gradually decreased relative to the initial drug release. At the end of the third month, it was determined that the drug release had decreased significantly under all of the storage conditions mentioned. This was due to a significant rise in the preparation viscosities under the same circumstances. However, no apparent increase in drug release was seen throughout or at the end of the second month. Due to the evaporation of the vehicle and a decrease in the total volume of sol, the viscosity significantly increased towards the end of the third month. The evaluation parameters under each of the specified storage conditions are shown in Table 4.

### 2.10. Differential Scanning Calorimeter (DSC)

**Figure 5:**
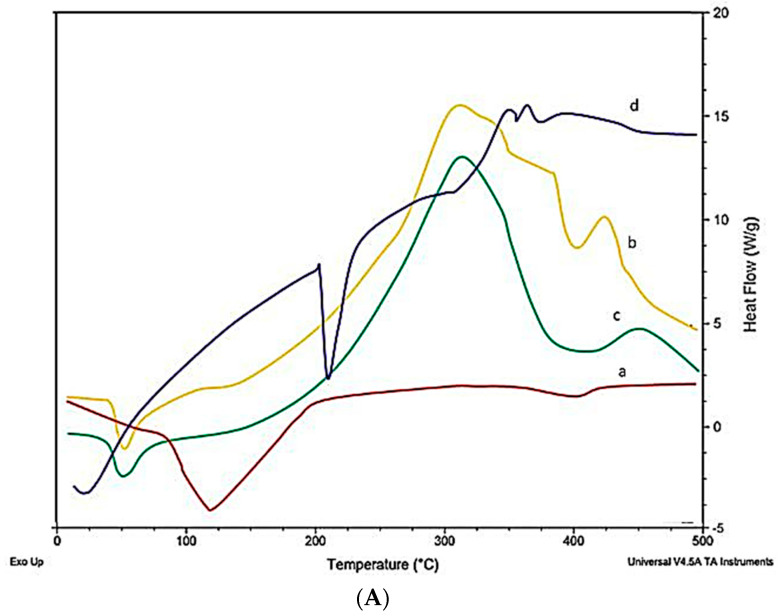

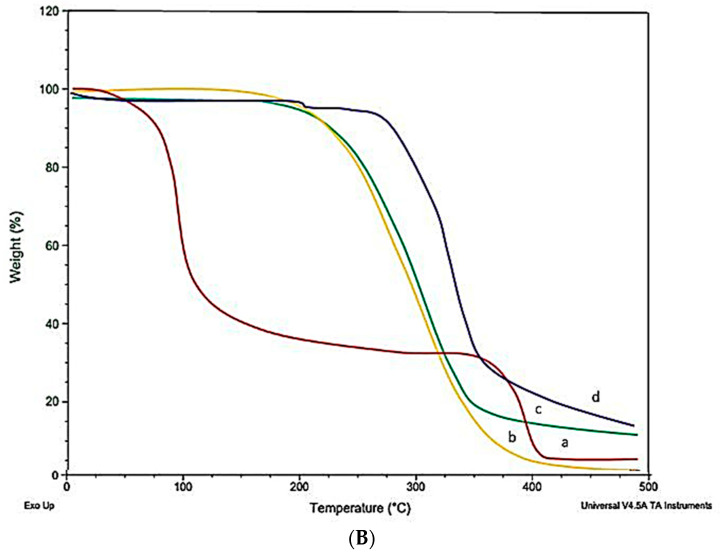
(**A**) DSC and (**B**) TGA: (a) LTG active, (b) poloxamer 407, (c) poloxamer 188, (d) LTG sol–gel.

LTG usually shows an intense endothermic peak at 218°, indicating the melting point and crystalline nature of LTG. Figure 5 shows the DSC and TGA of the drug, polymers, and LTG sol–gel. The strong endothermic peak of LTG disappeared in the LTG sol–gel, indicating that LTG was present inside the polymer matrix and that it had been changed from a crystalline to an amorphous form. Glycerin and polymers present in the formulation act as plasticizers and shrink the melting point of the formulation.

### 2.11. X-ray Diffraction (XRD)

**Figure 6:**
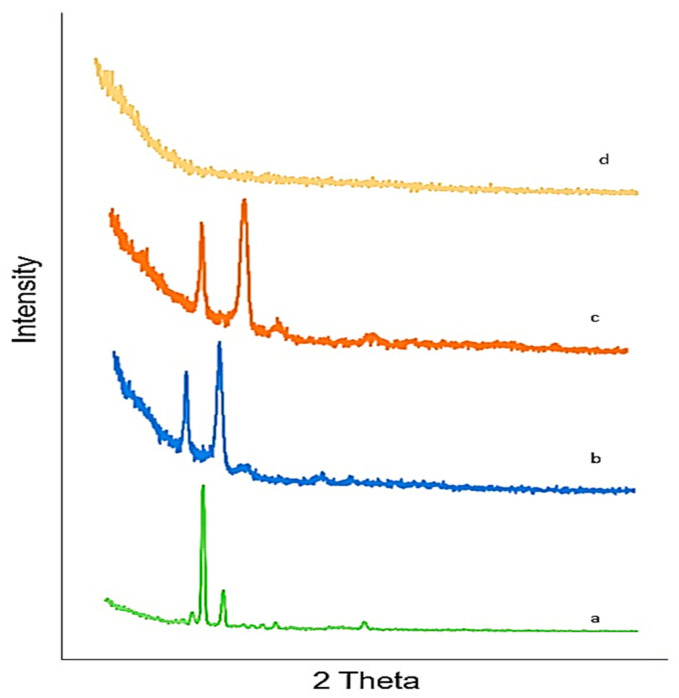
XRD: (a) LTG active, (b) poloxamer 407, (c) poloxamer 188, (d) LTG sol–gel.

Lamotrigine exhibits sharp peaks between 10° and 30°, with 2Θ indicating the crystalline nature of lamotrigine LTG sol–gel. The sharp peak of LTG usually present in pure LTG disappeared, suggesting the amorphous nature of the LTG sol–gel. The drug was diffused in the polymer matrix. Figure 6 displays the X-ray diffraction pattern of the drug, polymers, and LTG sol–gel.

### 2.12. Fourier Transform Infrared Spectrophotometer (FTIR)

#### Lamotrigine Sol–Gel and Polymers

**Figure 7:**
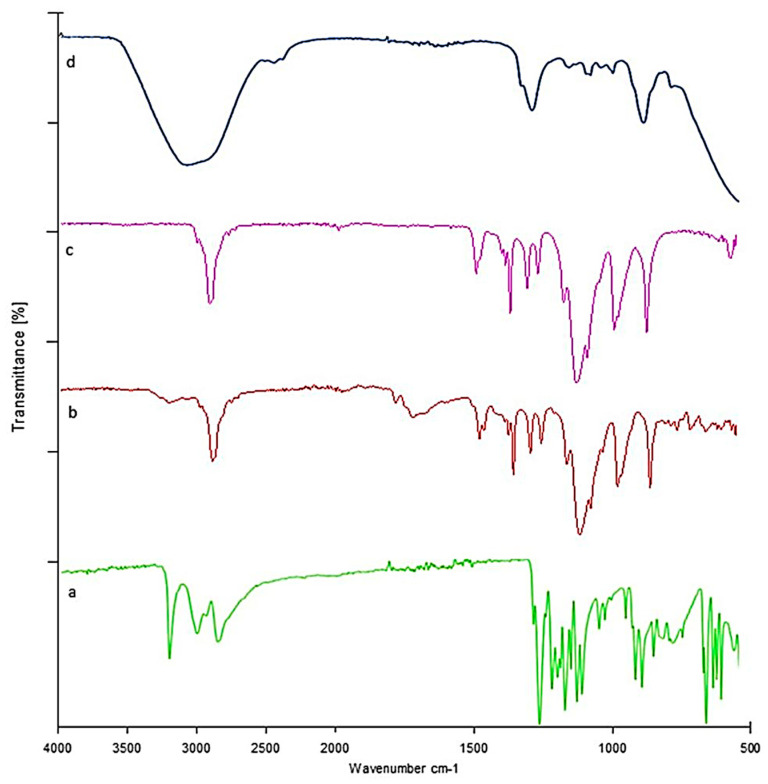
FTIR: (a) LTG sol-gel, (b) poloxamer 188, (c) poloxamer 407, (d) LTG sol-gel.

Pure LTG FTIR chromatograms showed distinctive peaks at 716, 791, 1140, 1617, and 3446 cm^−1^, which have been found to correspond to the stretching of C=H, C-Cl, C-N, C=C, and N-H, respectively. These distinctive peaks were still present in the LTG sol–gel, which indicates that our drug and the excipients were compatible. This was confirmed by the existence of LTG characteristic peaks and the lack of additional peaks in LTG thermos-responsive sol–gel. Figure 7 shows the FTIR chromatogram of the drug and polymers.

### 2.13. Drug Release Studies In Vitro

**Figure 8:**
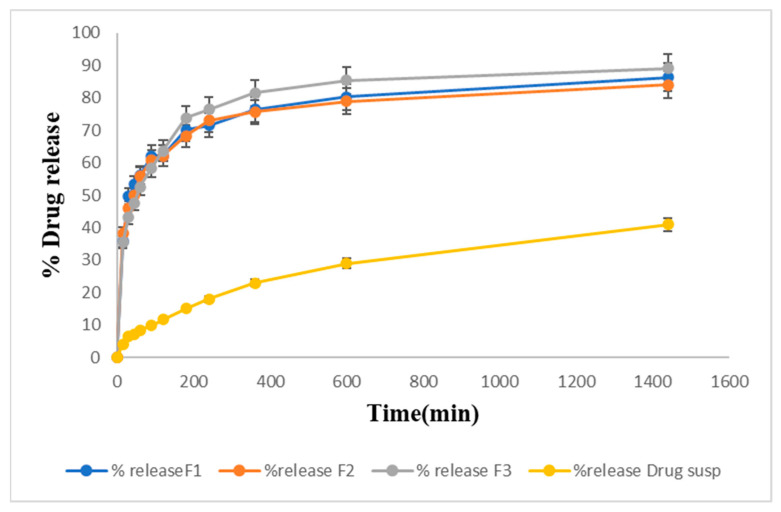
Drug release studies from prepared LTG sol–gel and drug suspension graphically.

An in vitro drug release study was performed on formulations F1, F2, and F3 and the pure drug suspension in PBS pH 6.4. Figure 8 shows the percentage of drug release of the formulations. F3 (92%) exhibited the maximum drug release. F2 showed 87% drug release, and F1 showed 84% drug release within 24 h. The percentage of drug release from the pure drug suspension was found to be 41%. LTG is a BCS class II drug with low aqueous solubility. Poloxamer 407 used in the formulation enhances the solubility of lamotrigine because it has both hydrophilic and hydrophobic characteristics.

#### Drug Release Kinetic Study

The dissolution profile is described by many mathematical functions that form the foundation of model-dependent approaches. Once an appropriate function was chosen, the generated model parameters were used to evaluate the dissolution profiles. First-order, zero-order, Higuchi, Hixson–Crowell, Korsmeyer–Peppas, were among the model-dependent methods [25]. After applying kinetic modeling to three of the selected formulations, the results obtained are shown in Table 5. The best-fitted model for in vitro drug release data was the Korsmeyer–Peppas model. The value of n in this model describes the drug’s release mechanism, which was n = 0.2. As the value of n was less than 0.5, our drug release followed the Fickian diffusion mechanism.

### 2.14. In Vitro Permeation Studies

**Figure 9:**
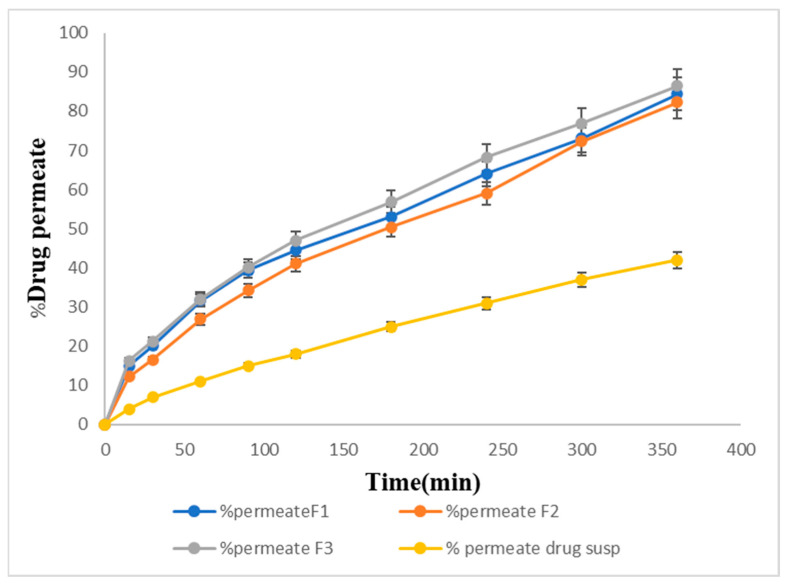
In vitro permeation percentage of the prepared formulations.

Figure 9 shows the percentage of drug permeation of the formulations F1, F2, and F3 through the membrane. F1 showed 80% permeation, F2 showed 78% permeation, and F3 (82%) showed the maximum drug permeation within 6 h. The pure drug suspension showed the lowest drug permeation, i.e., only 42% within 6 h.

### 2.15. Ex Vivo Permeation of Sol–Gel

**Figure 10:**
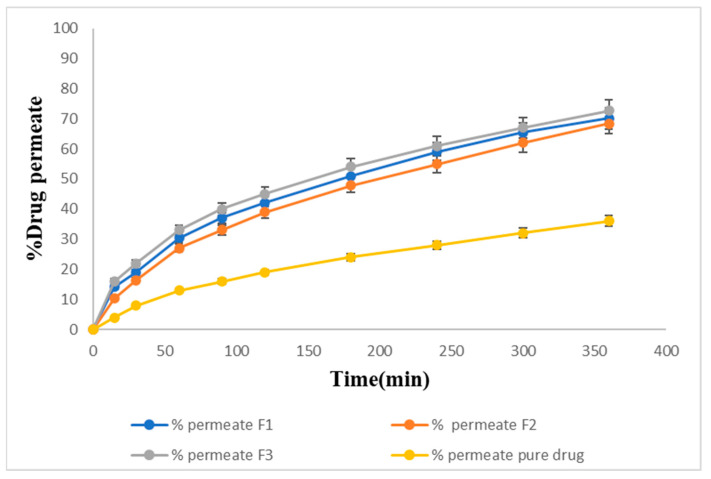
Ex-vivo permeation percentage of the prepared formulations.

The ex vivo permeation studies showed slightly slower release for the same formulations. F1, F2, and F3 showed 70%, 68%, and 72% drug permeation, respectively, within 6 h, and 36% of the drug was permeated from the pure drug suspension through the goat nasal mucosa at the end of 6 h. The maximum percentage of the drug, 72%, was permeated from F3 as shown in Figure 10.

### 2.16. Acute Toxicological Study

**Figure 11:**
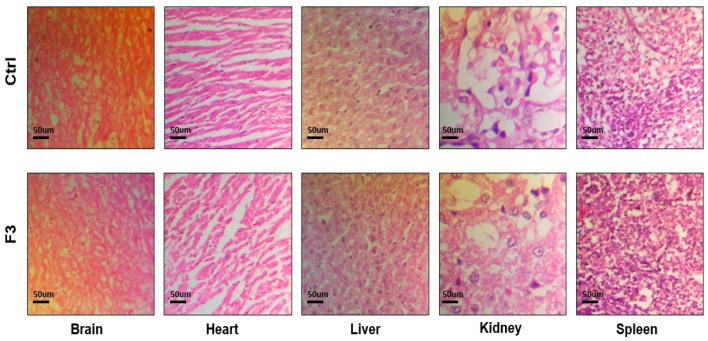
Histological analysis of control and sol–gel-treated groups.

**Figure 12:**
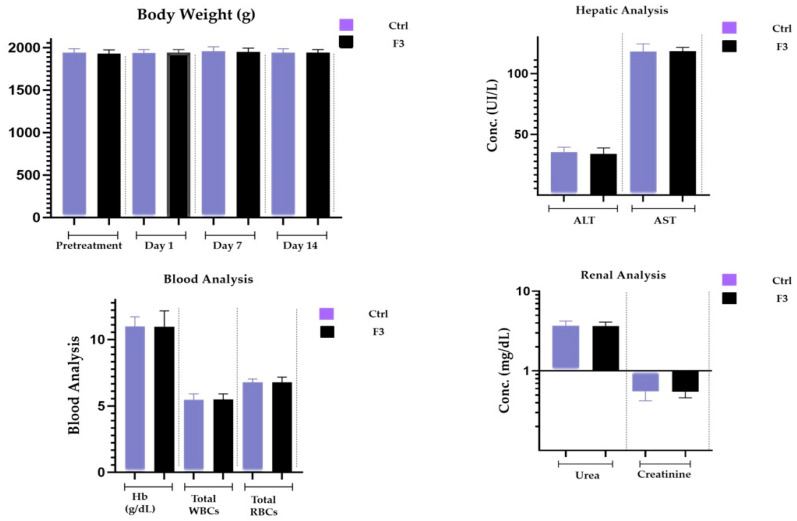
Body weight, renal, hepatic, and blood analyses.

Both the control group and the sol–gel-treated group underwent microscopic tissue analysis. The heart, liver, spleen, kidney, and brain all lacked any abnormal lesions. Both groups’ absolute organ weights were within the normal range. Figure 11 illustrates that none of the aforementioned essential organs had any major abnormalities overall and showed no signs of degeneration. There was no variation in body weight in the treated group. The hepatic analysis showed normal ALT and AST levels (Figure 12). The results of the renal analysis indicated normal urea and creatinine clearance. There was no abnormality seen in the blood analysis report.

## 3. Discussion

In this study, a total of ten formulations of LTG sol–gel was formulated, and the three best out of ten were selected for further testing and assessment, i.e., pH, gelation temperature and time, drug content, viscosity, drug content, spreadability, in vitro drug release, FTIR, differential scanning calorimetry, X-ray diffraction, ex vivo permeation studies, and in vivo toxicological studies. Our main objective was to formulate a temperature-responsive sol–gel with a transition temperature below 350 °C. An optimized formulation in solution form has a clear appearance at room temperature, which is an ideal characteristic of sol–gel, and is turned into gel at 310 °C. The pH of the optimized formulation lies within the pH range of the nasal mucosa (5.5 to 6.5). When the pH is near the pH of the nasal mucosa, the drug shows better absorption and prevents irritation after nasal administration. LTG sol–gel was optimized by 2^3^ factorial designs to select the best combination of polymers, poloxamer 407 and poloxamer 188, and also to achieve the desired gelation temperature and gelling time of the dosage form. A formulation containing PF127 (20% *w*/*v*), PF68 (13% *w*/*v*), and glycerin 2% in combination was selected as the optimized formulation.

The quantity of polymers present determined the nature of the gel that was formed. The temperature that turns sol into gel is the gelling temperature. If the gelling temperature is lower than the reference period, a gel can form at room temperature. However, when the gelation temperature rises, nasal mucosal gelation does not take place, resulting in quick nasal clearance. Gelling time is a measure of the time it takes for a liquid to turn into a gel after administration in the nasal cavity. Our formulation was converted into gel within 30 s. It was observed that the concentrations of poloxamers 407 and 188 had a linear effect on the sol–gel temperature at which gelation occurred. The test results showed that the sol–gel transition temperature decreased upon increasing the concentration of Pluronic F127, and the sol–gel transition temperature decreased by lowering the concentration of Pluronic F68. The consistency of the formulation was improved as the concentration of poloxamer 407 increased. This will lead to better drug entrapment and prevent drug leakage. To enable sustained local drug release, the in situ gel should maintain its integrity for a longer period without dissolving or degrading. It was found that as the poloxamer 407 concentration increased, the viscosity of the formulation also increased. A value of gel strength between 25 and 50 s is considered appropriate. Gels with a strength of less than 25 s have a risk of losing their integrity and eroding quickly, while gels with a strength of more than 50 s are excessively stiff and could cause irritation to the mucosal membranes. A higher concentration of polymers directly increased the stiffness of the formulation. Spreadability was satisfactory for all formulations. The drug content before and after gelation showed no significant change, indicating dose uniformity in the formulations. According to FTIR analysis, the distinctive peaks of LTG were still present in LTG sol–gel, indicating the compatibility of the drug with the other excipients present in the formulation. DSC/TGA analysis indicated that there was no major shift seen, but there was a slight shrinkage of the melting point due to poloxamers and glycerin, which act as plasticizers. The sharp peaks present in the XRD of active drug are due to the crystalline nature of LTG, which disappeared in LTG sol–gel, indicating the amorphous nature of LTG sol–gel. In vitro, a drug release study of all three formulations was performed, and for comparative analysis, an in vitro drug release study of pure drug suspension was also performed to check the effect of poloxamer 407 on the solubility of LTG. Within 24 h, 92% of the drug was released from the LTG sol–gel, and 41% of the drug was released from the pure LTG suspension. To determine the mechanism of drug release, the in vitro dissolution data of lamotrigine formulations were submitted to a goodness of fit test using linear regression analysis by zero-order and first-order kinetic equations, Higuchi’s, and Korsmeyer–Peppa’s models. Drug release data were best fitted with the Korsmeyer–Peppas model, which had an R^2^ of 0.99 and a value of n < 0.5. The mechanism that reports the release of actives from the polymer matrix is indicated by the value of n. The drug release kinetics followed the Fiskian diffusion-controlled drug release mechanism. In vitro permeation studies demonstrated that LTG has high permeability, which makes it effective for direct nose-to-brain delivery. Ex vivo permeation studies showed slightly slower permeation through the goat nasal mucosa. LTG sol–gel showed high permeability compared to a pure drug suspension. Poloxamer 407, present in LTG sol–gel, also acts as a permeation enhancer. To attach to the nasal mucosa, poloxamer 407 forms hydrogen bonds. Additionally, poloxamer 407 works as an inhibitor of P-gp (the efflux pump), which makes it easier for LTG to permeate [26]. An acute toxicological study was performed on Albino rabbits. A dose of 0.1 mg/kg was administered in the nostrils of rabbits alternately for two weeks, and the dose was increased moderately to 10 mg/kg. No physical damage or inflammation of the nasal mucosa was seen. The results indicated no signs of toxicity or degeneration.

## 4. Conclusions

It was discovered that the development of an LTG thermosensitive sol–gel was well suited for intranasal administration to produce a quick onset of action and extend the drug’s nasal residence period. The gel’s thermo-reversible properties made it easier to administer and handle. The optimized formulation, which contains the active ingredients PF127 (20% *w*/*v*), PF68 (13% *w*/*v*), and glycerin (2% *w*/*v*)*,* is safe for routine nasal administration, stable at 25 °C and 4 °C, suitable for the desired phase transition temperature of 34 °C, and has appropriate viscosity and gel strength. Thus, it is concluded that the intranasal route could prove to be the most effective drug delivery method for managing epilepsy, which could lead to issues with pharmaco-resistance resulting from conventional epileptic therapy.

## 5. Materials and Methods

### 5.1. Materials

Lamotrigine was received as a gift sample from STANDPHARM, (Pvt) Ltd., Lahore, Pakistan. Poloxamer 407 (Pluronic F127) and poloxamer 188 (Pluronic F68) were purchased from Sigma-Aldrich Germany. Distilled water was produced in a post-graduate research lab at the University of Central Punjab, Lahore, Pakistan, during the research. Ethanol and acetone were purchased from Sigma Aldrich, Darmstadt, Germany, and glycerin was purchased from Flav Chem Royal, Shanghai, China.

### 5.2. Method to Prepare Thermo-Responsive Sol–Gel of Lamotrigine

Lamotrigine sol–gel was prepared by using the cold method [27,28]. Poloxamer 407, poloxamer 188, and glycerin were mixed in the required quantity of distilled water and stirred using a magnetic stirrer for 1 h at 200 to 300 rpm to avoid further formation. After stirring, this mixture was refrigerated overnight at 4 °C until a clear solution was obtained. After 24 h, lamotrigine was dissolved in ethanol and acetone and then slowly added to the prepared base while stirring and mixed with continuous stirring for 1 h. Furthermore, it was adjusted to pH 6.4. The gelation temperature was determined by checking visually. Ten formulations were prepared by varying the concentrations of poloxamer 407 and poloxamer 188, as shown in Table 6 [29]. Table 1 represents the composition and percentage of the polymers and excipients used in the formulations.

### 5.3. Characterization and Evaluation

#### 5.3.1. Formulation Optimization

Design Expert 12 is the software that was used to optimize the formulations. The main effects and interactions of the poloxamers 407 (X1) and 188 (X2) at different concentrations were identified using a central composite design of a response surface methodology. To choose the best formulation, the sol–gel transition temperature was used as an indication. The sol–gel transition temperatures obtained varied from 28 °C to 38 °C and were calculated from 10 experimental runs produced by the central composite design by fitting the response surface model. Different poloxamers 407 and 188 were used in the optimization process, which was carried out using a two-factor, three-level factorial design. The statistical experimental design was created and assessed using Design-Expert^®^ software, version 14 [30]. In Table 1, several trials have been generated and described as coded levels. Table 7 discusses the associated values for formulation compositions.

After putting the data within the polynomial equation (Equation (1)), an analysis of variance (ANOVA) was carried out employing a 95% confidence interval: [31]

Y = β_0_ + b1X_1_ + b_2_X_2_ + b_12_X_1_X_2_ + b_1_X_1_^2^ + b_2_X_2_^2^ (3)

The arithmetic mean response of running trials is β_0_, and the dependent variable is represented by Y. In this study, X_1_ and X_2_ were two variables that were changed from low to high values. A positive or negative result was judged by a polynomial equation.

#### 5.3.2. Clarity

The clarity of the sol–gel was assessed visually against a dark background [32].

#### 5.3.3. pH

The pH of the nasal mucosa should be between 5.5 and 6.5. The formulation pH provided by the intranasal route should be between 5.5 and 6.5. To measure the pH of the sol–gel formulation, 1 mL of each formulation was transferred into a new beaker and diluted with distilled water until it contained 25 mL. The pH of each formulation was then assessed using a pH meter (the ADWA pH meter (AD1050)) [33].

#### 5.3.4. Viscosity Measurement

The viscosity of the intranasal sol–gel was evaluated using a Brookfield viscometer (BDV-8S). Before taking the measurements, the sample was equilibrated for 10 min. The viscosity of the sol–gel was measured by transferring the prepared formulation into the beaker, and spindle no. 3 was dipped into the beaker of the gel at a specific angle of 60 rpm and maintained at 37 °C. The viscosity of in situ gels should be under 16,000 cps [34].

#### 5.3.5. Sol–Gel Gelling Temperature and Time Measurement

The term “gelation temperature” means that upon tilting, the meniscus of the test tube is not moved. Miller and Donovan’s technique describes the gelation temperature. According to this technique, 2 mL of prepared sol–gel was transferred into the glass vial and placed in a water bath at 8 °C, and then the temperature of the water bath was increased slowly from 30 °C to 35 °C by an increment of 2° to 3° when started and then by 2° to 5° until gelation. After each adjustment of the water bath, the gel took 5 min to equilibrate. Then, the formulation was checked for gelation [35,36].

#### 5.3.6. Gel Strength

The sample (5 g) was placed in a glass vial with a capacity of 20 mL. To gel the formulations, the thermostat setting was kept at 35 °C. To determine the gel’s strength, a time duration of 3.5 g to sink 5 cm into the gel was used.

#### 5.3.7. Drug Content

A modified UV-Vis spectroscopic technique was used to determine the uniformity of the drug concentration prior to and after gelling [37,38]. First, 1 mL of the prepared formulation was dissolved in 10 mL of methanol properly. The resultant mixture was filtered through a filter paper size of 0.45 µm and then diluted with PBS pH 6.4. The amount of lamotrigine in the formulation was determined using a spectrophotometer at 307 nm (Shimadzu 1800, Japan).

#### 5.3.8. Spreadability

A rectangular glass slide measuring 10 × 4 cm was used to measure the spreadability. A thread was used to secure the artificial membrane to the slide’s surface. One drop of gel was applied to it at an angle of 120° while the slide was heated to 35 °C in a hot air oven. Spreadability was assessed in relation to the distance a drop of liquid gel traveled before gelating. Three readings on average were taken [39].

#### 5.3.9. Stability Studies

In accordance with the requirements of the International Conference on Harmonization, stability studies were conducted on the sol–gel formulation. Sol–gel bottles were kept at room temperature with a relative humidity (RH) of 65% and at 4 °C for 3 months. After 3 months, changes in the stored formulations in terms of their appearance, viscosity, drug content, and in vitro drug release were checked [40].

#### 5.3.10. Differential Scanning Calorimeter

When using the thermal analysis technique known as differential scanning calorimetry (DSC), a sample is subjected to a controlled temperature program while the heat flow into or out of the sample is monitored as a function of temperature or time [41,42]. In this study, DSC was used to evaluate the thermal behavior of lamotrigine in its formulation and in its pure form. In total, 10 mg of samples was weighed, sealed in common aluminum pans, and then heated at a rate of 10 C/min while being scanned throughout a temperature range of 50 °C to 300 °C [43].

#### 5.3.11. X-ray Diffraction (XRD) Analysis

X-ray diffraction analysis (XRD) is a technique that provides complete information about the crystalline structure, physical properties, and chemical composition of a material [44]. XRD analysis was conducted to analyze the structure of the polymers used and the prepared nanoparticle formulations for their degree of crystallinity. This helped to evaluate the impact of changes on the crystallinity of formulations [45].

#### 5.3.12. Zeta Potential

Zeta potential analysis is a useful tool for determining the surface charge of particles, and it is closely related to the preparation composition. It has been proven to have a significant impact on the stability of colloidal dispersion, nanoparticles, nano-emulsions, and other materials. This demonstrates that flocculation or aggregation is forbidden, which results in the development of a formulation with an extended shelf life. The zeta potential, which is primarily responsible for the stability of the colloidal dispersions, was used to determine the surface charge of the particles [46,47].

#### 5.3.13. FTIR (Fourier Transform Infrared Spectrophotometer)

The Fourier transform infrared spectrophotometer (FTIR) is the most important tool for identifying the various types of chemical bonds or functional groups [48]. The range of wavelength of the FTIR instrument is 10,000 to 100 cm^−1^. The sample is exposed to infrared light; the sample absorbs some light, some of which passes through. The sample molecules transform the absorbed energy into rotational or vibrational energy. The resultant signal appears as a spectrum at the detector and generally ranges from 4000 cm^−1^ to 400 cm^−1^, representing the sample’s molecular fingerprint. Because each molecule or chemical structure will provide a distinct spectral fingerprint, FTIR analysis is a fantastic technique for identifying specific chemicals. The spectrum was recorded on a FTIR instrument (Perkin Elmer Spectrum Two, Waltham, Massachusetts, USA) using PC-based software-controlled instrument operation and processing of the data [49].

#### 5.3.14. Lamotrigine Calibration Curve

##### Preparation of a Standard Stock Solution

To prepare a standard stock solution, 10 mg of lamotrigine was dissolved in 10 mL of methanol (1 mg/mL). Then, 1 mL of this solution was poured into 10 mL of the buffer of pH 6.4 [50] to make the strength of 1000 μg/mL. Then, from this solution, dilutions of concentrations of 5 µg/mL, 10 µg/mL, 15 µg/mL, 20 µg/mL, and 40 µg/mL were made [51].

##### Formation of a Calibration Curve

First, 0.5 mL of stock solution was taken out and then diluted with PBS of pH 6.4 to make up a total volume of up to 10 mL to make the concentration of 5 μg/mL. Then, 1 mL of solution was taken and diluted in buffer to make the final volume up to 10 mL to make a concentration of 10 μg/mL. Again, 1.5 mL of solution was taken and diluted in buffer 8.5 mL to make a concentration of 15 μg/mL. Again, 2 mL of solution was taken and diluted in buffer 8 mL to make a concentration of 20 μg/mL. Then, at last, 4 mL of solution was taken and diluted in 6 mL of buffer to make a concentration of 40 μg/mL. The absorbance of a solution containing a concentration of 5 to 40 μg/mL was determined using a UV/visible spectrophotometer (LC 95, Perkin Elmer, Waltham, MA, USA) at a wavelength of 307 nm. The graph was plotted using absorbance versus concentration, and the values of R^2^, slope, and intercept were calculated using a linear equation [16,52].

#### 5.3.15. In Vitro Drug Release of Lamotrigine Sol–Gel

By using the USP Dissolution Apparatus-II at 35 °C and 50 rpm, a drug release study was performed. The PBS (pH 6.4) was filled in the apparatus’s vessels, and 1 mL of each formulation was added to the dialysis membrane as it was tied up with thread at one end. The dialysis membranes were filled with 1 mL of formulations, knotted at both ends, and then 2 mL of the sample was taken out after 5, 1, 35, 10, 30, 45, 60, 90, 120, 180, 240, 360, and 600 min. At the same time, the volume takeout was replaced by fresh PBS (2 mL). The concentration in the sample was detected using a UV spectrophotometer at a wavelength of 307 nm [53,54].

#### 5.3.16. Drug Release Kinetic Study

The drug release constants and regression coefficients (R^2^) were calculated with the help of DDsolver software version 10. Kinetic modeling was performed by applying different models, such as first-order, zero-order, Higuchi, and Korsmeyer–Peppas, to in vitro drug release data in order to determine the mechanism of drug release from sol–gel.

#### 5.3.17. In Vitro Permeation of Sol–Gel

Franz diffusion cells were used to measure the in vitro permeation of lamotrigine. The artificial dialysis membrane was placed between the donor and the receptor. Phosphate buffer (pH 6.4) was added to the receptor compartment at a temperature of 37 °C. At 100 rpm, the solution swirled. The compartments were clamped together after the gel (10 mg) was applied to the membrane. The Franz diffusion cell was used for in vivo permeation studies. One (1) mL of sample and seven (7) mL of buffer were added to the donor compartment and the recipient compartment, respectively. The cellophane membrane was left open, allowing the medication to pass through. Then, 1 mL of each sample was taken out at various time intervals (15, 30, 60, 90, 120, 180, 240, 300, and 360 min), and the volume was replaced with 1 mL of PBS pH 6.4 and subjected to UV examination at 309 nm to determine how much of the drug had permeated.

#### 5.3.18. Ex Vivo Permeation of Sol–Gel

Goat nasal mucosa was selected for the ex vivo permeation study. From the nasal cavity, the fresh mucosal layer was separated from a goat head that was collected from a local slaughterhouse and used within 4 h of the animal’s slaughter. The mucosal layer was hydrated using a saline solution throughout the procedure. PBS (pH 6.4) was filled in the receptor compartment (7 mL) of the Franz’s cell. The mucosal layer was mounted on a dialysis tube and secured between the donor and receptor chambers using clamps, with the mucosal side facing the donor and a diffusion area of 0.63 cm^2^. The temperature of the medium was kept at 35 °C. The LTG sol–gel (1 mL) was poured into the donor compartment. Then, 1 mL of each sample was withdrawn from the receiver compartment after 15, 30, 60, 90, 120, 180, 240, 300, and 360 min, and the volume was replaced with 1 mL of PBS pH 6.4 and subjected to UV examination at 307 nm to determine how much of the drug had permeated.

#### 5.3.19. Acute Toxicology Study

The acute toxicology study of thermo-responsive lamotrigine sol–gel was assessed using the Maximum Tolerated Dose Method. Albino rabbits (weighing between 800 and 10,200 g) of both sexes were bought from the animal laboratory at Rifah University. All tests were carried out according to OECD recommendations, which were then confirmed by the UCP ethical committee (Ref. No. UCP/ORIC/TDF/App#10/2023). Male rabbits (n = 8) were housed in a clean environment with a 12 h light/dark cycle and fed a regular meal and water. LTG sol–gel (0.1 mg/kg BW) was given by intranasal route (50 µL/nostril) to the treatment group only using an intubation cannula. Doses were increased moderately from 0.1 mg/kg to 10 mg/kg. All information related to signs of toxicity, poor health, death, and any other activity consequence was recorded twice daily for two weeks in all animals. After two weeks, tests for blood, clinical biochemistry, gross necropsy, and histopathology were performed. Overall, comparisons were made between the treatment group and the control group for each parameter.

To conduct various clinic–pathological examinations, plasma was isolated and analyzed. The following tests were run: ALT, AST, cholesterol, triglycerides, creatinine, urea, and uric acid. All of the rabbits were killed after two weeks. On many significant organs, including the heart, liver, spleen, kidney, and brain, we performed gross autopsies. We then determined the relative organ weights by weighing the various organs. Each organ was kept in a 4% buffered formalin solution for storage. Selected tissues were paraffin-embedded and then sliced into 4–5 m thicknesses for hematoxylin–eosin staining and histopathological investigation.

## Figures and Tables

**Table 1:** Experimental design of independent (X1, X2) and dependent (R1, R2) variable parameters.

Run	(X1) Polox 407 (%)	(X2) Polox 188 (%)	(R1) Gelation Temp (°C)	(R1) Gelation Time (s)
1	20	13	31	40
2	19.5	15.5	35	56
3	18	15.5	35	56
4	19.5	12	28	35
5	20	15.5	34	52
6	19	13	33	45
7	19	18	37	59
8	20	16	34	52
9	19.5	15.5	35	56
10	20	18	36	57

**Table 2:** Analysis of variance for gelation temperature and gelling time.

Variables	Gelation Temperature °C	Gelling Time (s)
B_0_	34	54
A (poloxamer 407(A_1_))	−0.05	−1.76
B (poloxamer 188 (B_2_))	2.89	7.94
AB	0.68	0.75
A2	−0.05	0.0625
B2	−3.4	−4.06
Model *p*-value	0.004	0.003
R^2^	0.89	0.99
Adjusted R^2^	0.86	0.98
F Value	29.54	101

**Table 3:** pH, viscosity, gelling temperature, gelling time of drug content a/f gelation, gel strength, and spreadability of lamotrigine sol–gel.

Formulations	pH	Viscosity (cp)	Gel Temp (°C)	Gel Time (s)	Gel Strength (s)	Drug Content (%)	Spreadability (cm)
F1	6.2	15,000	36	57	55	93	7
F2	6.5	14,780	34	52	45	90	9
F3	6.4	14,000	31	40	35	94	12

**Table 4:** Stability studies.

Evaluation Parameter	at 4–8 °C		
	1st Month	2nd Month	3rd Month
In vitro drug release (%)	92.05	91.34	90.45
Viscosity (cp)	14,000	14,100	14,212
Appearance	No change	No change	No change
**Evaluation Parameter**	**at 25 °C**		
	**1st Month**	**2nd Month**	**3rd Month**
In vitro drug release (%)	92.05	89.34	88.45
Viscosity (cp)	14,000	14,560	15,000
Appearance	No change	No change	No change

**Table 5:** Kinetic modeling for drug release through sol–gel.

In Vitro Drug Release				
Kinetic Models		F1	F2	F3
Zero-order	K^0^	0.093	0.092	0.097
	R^2^	3.00	0.035	2.30
First-order	K^1^	0.011	0.011	0.010
	R^2^	0.491	0.47	0.738
Higuchi model	kH	3.527	3.471	3.632
	R^2^	0.29	0.303	0.032
Korsmeyer–Peppas model	kKP	29	28.7	30
	R^2^	0.97	0.975	0.99
Best-Fit Model		Korsmeyer–Peppas	Korsmeyer–Peppas	Korsmeyer–Peppas

**Table 6:** Coded level of RSM.

Name	Units	Low	High	−Alpha	+Alpha
Polox 407	%	19	20	18.7929	20.2071
Polox 188	%	13	18	11.9645	19.0355

**Table 7:** Composition of trial formulations.

Trial	Polox 407 (%)	Polox 188 (%)
F1	20	13
F2	19.5	15.5
F3	18	15.5
F4	19.5	12
F5	20	15.5
F6	19	13
F7	19	18
F8	20	16
F9	19.5	15.5
F10	20	18

## Data Availability

All of the data are contained in the manuscript.
